# Tick-borne agents in the fowl tick *Argas persicus* from northwest and northeast China

**DOI:** 10.1186/s13071-025-06750-x

**Published:** 2025-04-19

**Authors:** Junhua Tian, Jing Liu, Kun Li, Li Zhong, Miao Lu, Hai Jiang, Runda Jie, Xiao Wang, Bing Zhang

**Affiliations:** 1 Wuhan Center for Disease Control and Prevention, Wuhan, 430024 Hubei China; 2 National Institute for Communicable Disease Control and Prevention, Chinese Center for Disease Control and Prevention, Changping District, Beijing, 102206 China; 3 Xinjiang Key Laboratory of Molecular Biology for Endemic Diseases, School of Basic Medical Sciences, Institute of Medical Sciences of Xinjiang Medical University, Xinjiang Medical University, Urumqi City, 830011 China; 4 Xinjiang 474 Hospital, China RongTong Medical Healthcare Group CO.LTD, Urumqi, 830011 Xinjiang Uygur Autonomous Region China

**Keywords:** *Argas persicus*, *Rickettsia hoogstraalii*, *Rickettsia* sp., *Coxiella*

## Abstract

Although tick-borne agents have been extensively studied, etiological investigations on soft ticks are still relatively rare. In this study, we collected 114 *Argas persicus* ticks from two provinces (Xinjiang and Heilongjiang) located in northwest and northeast China, respectively, and screened them for tick-borne agents. Two *Rickettsia* species were identified in *A. persicus* ticks from Heilongjiang Province: *Rickettsia hoogstraalii* (27.3%, 18/66) and a previously unidentified species (12.2%, 8/66). The 16S rDNA, *gltA*, *groEL*, and *ompB* genes of the latter have 98.8%, 93.1%, 94.3%, and 91.2% nucleotide identities to reported species, suggesting that it represents a novel species. It belongs to the ancient group of *Rickettsia* and is located in the basal position of the phylogenetic trees. Additionally, *Coxiella* endosymbiont was detected in *A. persicus* ticks from both locations with 100% positive rates. Furthermore, the *Coxiella* endosymbionts from different locations form distinct phylogenetic groups, indicating that one tick species can harbor different *Coxiella* endosymbionts.

**Graphical abstract:**

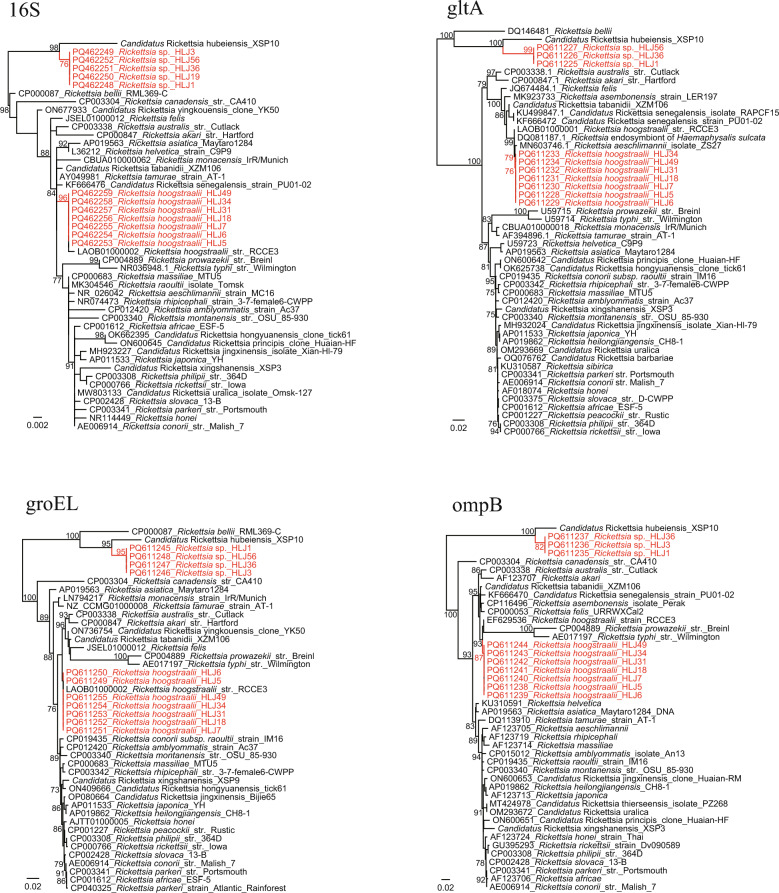

**Supplementary Information:**

The online version contains supplementary material available at 10.1186/s13071-025-06750-x.

The fowl tick *Argas persicus* (Ixodida: Argasidae) is a soft tick commonly associated with poultry [1]. Although its major natural hosts are pigeons, swallows, and chickens, it is sometimes found on domestic animals such as sheep and cattle [2]. It is also reported to attack humans (especially in early Persia), making it possible to transmit pathogens from animals to humans [3]. Tick-borne agents (e.g., *Rickettsia*, *Anaplasma*, *Coxiella*, *Borrelia*, *Babesia*, Crimean-Congo hemorrhagic fever virus, and thrombocytopenia syndrome virus) have been extensively studied in China [4]. However, most investigations only focus on hard ticks (family Ixodidae), probably because of their easy availability. Although soft ticks are widely spread in China, their associated agents have been rarely reported. Herein, we collected *A. persicus* ticks in northwest and northeast China and studied the tick-borne agents in them.

From 2012 to 2017, soft ticks were collected in two locations: the 170 Regiment of the 9th Division in Xinjiang Uygur Autonomous Region, northwest China, and Hegang City in Heilongjiang Province, northeast China. A total of 48 ticks were collected in chicken coops in Xinjiang in the spring of 2012, and 66 ticks were collected in cattle sheds in Heilongjiang in the spring of 2017. The ticks were carefully collected using tweezers. Using taxonomic keys [5], all the ticks were morphologically identified as adult *A. persicus* (Additional file 1: Figure S1). The genomic DNA of the ticks was extracted using the EZNA® Mollusc & Insect DNA Kit (Omega) according to the manufacturer's instructions. The tick species was confirmed by amplifying and analyzing a 674-bp fragment of the *COI* gene as described elsewhere [6]. The *COI* sequences of ticks from Xinjiang were 100% identical to *A. persicus* isolate E5 (OM368319.1), while the sequence of ticks from Heilongjiang was 100% identical to *A. persicus* isolate H1 (OM368320.1). Tick-borne agents, including *Rickettsia*, Anaplasmataceae bacteria, *Coxiella*, *Borrelia*, *Bartonella*, and *Babesia*, were screened using primers as previously shown [7, 8].

All the DNA samples were negative for Anaplasmataceae bacteria (including *Ehrlichia*, *Anaplasma*, and *Neoehrlichia*), *Borrelia*, *Bartonella*, and *Babesia*. Two *Rickettsia* species were detected and initially identified by amplifying and analyzing the partial 16S rDNA sequences (PCR products of approximately 900 bp) in *A. persicus* ticks from Heilongjiang, with positive rates of 27.3% (18/66) and 12.1% (8/66), respectively. Subsequently, the partial *gltA* (PCR products of approximately 1000 bp), *groEL* (PCR products of approximately 1100 bp), and *ompB* (PCR products of approximately 650 bp) genes were amplified and sequenced from randomly selected samples, as described elsewhere [7]. Based on the results, one *Rickettsia* species was determined as *Rickettsia hoogstraalii*, with the 16S rDNA, *gltA*, and *ompB* sequences showing 99.9%, 99.8%, and 99.4% identities to *R. hoogstraalii* str. RCCE3 or str. Croatica (LAOB01000001, LAOB01000002, EF629536). Interestingly, the other species was genetically distant from all known *Rickettsia* species. The 16S rDNA sequences had 98.8% identity to *Rickettsia* endosymbiont of the box bug *Gonocerus acuteangulatus* (OZ032147.1), 98.8% to *Candidatus* Rickettsia hubeiensis (OR979082.1), and 98.6% to *Rickettsia bellii* strain RML369-C (NR074484.2). Similarly, the *groEL* and *ompB* sequences had the highest identities 94.3% and 91.2%, to *Candidatus* Rickettsia hubeiensis (*groEL*: OR971720.1, OR971721.1; *ompB*: PP146537.1, PP146538.1). For the *gltA* gene, the rickettsia was closely related to the *Rickettsia* endosymbiont of *Amblyomma patinoi* with a nucleotide similarity of 93.1% (MN817133.1). In the phylogenetic trees constructed using PhyML 3.0 with the GTR + I + G model, all the strains formed a distinct clade together with *R. bellii* and *Candidatus* Rickettsia hubeiensis (Fig. 1), suggesting that it represents a novel species in the ancient group of the genus *Rickettsia*. The criteria of a novel *Rickettsia* species proposed by Fournier et al. [9] also support our opinion that it is a novel species. No *Rickettsia* was detected in ticks from Xinjiang.

**Fig. 1:**
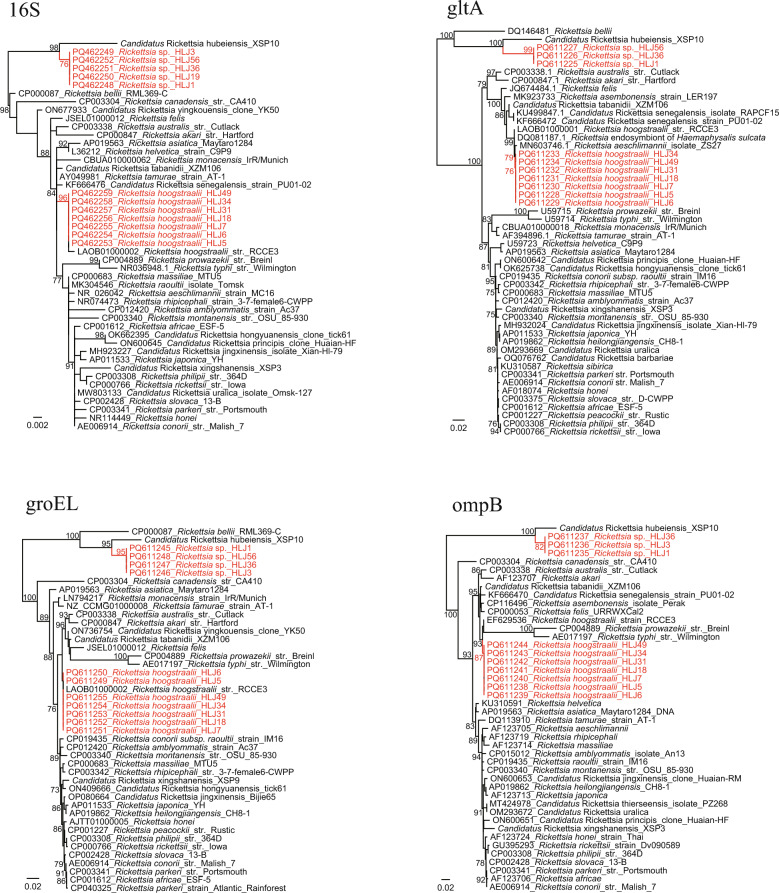
Phylogenetic trees based on the nucleotide sequences of 16S rDNA (809 bp), *gltA* (927–930 bp), *groEL* (1018 bp), and *ompB* (617 bp) genes of *Rickettsia* strains

*Coxiella* endosymbionts were detected using primers amplifying an approximately 550-bp conserved region of the *rpoB* gene [10]. All *A. persicus* ticks (positive rate 100%) were positive for *Coxiella*-like endosymbiont (CLE). However, in the phylogenetic tree based on *rpoB* sequences (Fig. 2), they were divided into two groups: one was only detected in ticks from Xinjiang (group 1), whereas the other was only detected in ticks from Heilongjiang (group 2). Both groups showed the highest nucleotide similarities to *Coxiella* sp. isolate EEZA-CRETAV2 identified in *Argas* sp. from Spain (MW287615.1) (96.1% and 98.7% nucleotide identities, respectively) and *Coxiella burnetii* (CP103427.1) (93. 9% and 95.6% nucleotide identities, respectively). For further exact identification of the CLEs, the *DnaK* sequence (PCR products of approximately 600 bp) was also obtained by nested PCR [10]. Similar to the *rpoB* gene, the *DnaK* sequences of CLE strains from ticks in Xinjiang and Heilongjiang were both closely related to *Coxiella* endosymbiont of *Ornithodoros rostratus* (KP985381.1) (with 96.2% and 97.1% nucleotide identities, respectively), *Argas monachus* (KP985358.1) (with 96.0% and 96.9% nucleotide identities, respectively), and *Coxiella burnetii* (ON455115.1) (with 95.2% and 95.4% nucleotide identities, respectively). In the phylogenetic tree, they also formed two distinct clades (Fig. 2).

**Fig. 2:**
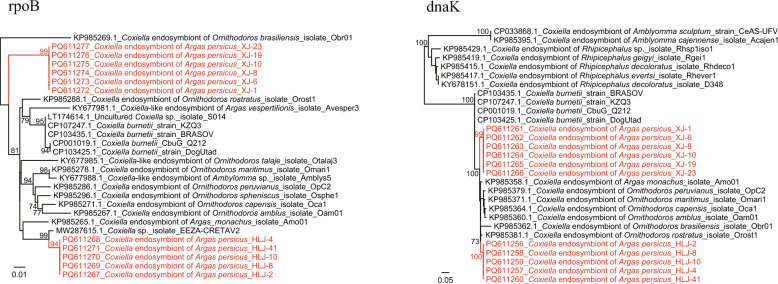
Phylogenetic trees based on the nucleotide sequences of *rpoB* (475 bp) and *dnaK* (560 bp) genes of *Coxiella* strains

In this study, two *Rickettsia* spp. were identified in *A. persicus* ticks from the same location in Heilongjiang Province: *R. hoogstraalii* and the novel species *Rickettsia* sp. *Rickettsia hoogstraalii*, belonging to the spotted fever group *Rickettsia* (SFGR), was first detected and isolated from *Haemaphysalis sulcata* in Croatia and *Carios capensis* in the USA, respectively [11]. Since then, it has been detected in multiple tick species (e.g., *Argas transgariepinus*, *Argas walkerae*, *Rhipicephalus rossicus*, *Hyalomma anatolicum*, *Ornithodoros faini*, *Haemaphysalis formosensis*) in several continents (Africa, Europe, Asia, and North America) [12–15]. In 2022, it was detected in *A. persicus* ticks from Gansu Province, Northwest China [16]. Our result confirmed the presence of *R. hoogstraalii* in China. Although it was only detected in ticks and never found in animals and humans, its close relationship with other SFGR members suggests its possible pathogenicity. Unexpectedly, a novel *Rickettsia* species belonging to the ancient group of genus *Rickettsia* was identified. In phylogenetic trees based on all genes investigated (i.e., 16S rDNA, *gltA*, *groEL*, and *ompB*), it was located in the basal position with *R. bellii* and *Candidatus* Rickettsia hubeiensis. Namely, it represents one of the several known members of the ancient group of *Rickettsia*. Until today, multiple *Rickettsia* species (e.g., *Rickettsia japonica*, *R. raoultii*, and *R. rickettsii*) have been identified in soft ticks [17, 18]. Of those, some were proposed as novel *Rickettsia* species. In 2018, a novel SFGR, *Rickettsia fournieri*, was identified in *Argas lagenoplastis* ticks in Australia [19]. As recently as 2025, a novel *Rickettsia* named "*Candidatus* Rickettsia vulgarisii" was described in *Argas* ticks from northwestern China [20]. However, almost all these *Rickettsia* spp. were SFGR, none belonging to the ancient group of *Rickettsia*. This result may contribute to knowledge of the genetic diversity of soft tick-borne *Rickettsia*.

CLE was detected in *A. persicus* ticks from both locations, with 100% positive rates, suggesting that CLE may be common in *A. persicus* ticks. Previous studies indicated that CLE is essential for B vitamin biosynthesis pathways in *Rhipicephalus*, *Amblyomma*, and *Ornithodoros* ticks [21]. Although the impact of CLE on the physiology of *Argas* ticks has never been studied, we suspect that it may play an important role in *A. persicus* considering the extremely high positive rates. Furthermore, it is interesting that the CLEs from different locations form distinct phylogenetic groups, indicating that one tick species can harbor different CLEs. They may have experienced long-term co-evolution together with their tick hosts. Besides, the *A. persicus* ticks from Xinjiang and Heilongjiang live in different habitats and have different animal hosts. They may have different sources of B vitamin intake. We suspect this might be one of the driving sources of CLE evolution.

In conclusion, we identified two *Rickettsia* species and two CLE species in *A. persicus* ticks from China. Of those, *R. hoogstraalii* belonging to SFGR may potentially infect animals and humans. Besides, although there are only a few reports that ancient group *Rickettsia* and CLEs infect animals or humans [22–24], the possibility remains. The presence of these agents in soft ticks may suggest a potential risk in these areas.

## Supplementary Information

Additional file 1: Fig. S1. Morphological characteristics of the *Argas persicus* tick from Xinjiang. The capitulum is not visible viewing from above. Body oval and flat dorso-ventrally. Discs are oval or rounded with different sizes. A lateral sutural line is present, with distinct rectangular squares around the entire body margin

## Data Availability

All sequences obtained in this study have been uploaded to the GenBank Database (accession nos. PQ462248–PQ462259, PQ611225–PQ611277).
