# Supplementary figures and images for: Tick-borne agents in the fowl tick Argas persicus from northwest and northeast China
Source: Parasit Vectors. 2025 Apr 19;18:145. doi: 10.1186/s13071-025-06750-x (PMC12009524; doi:10.1186/s13071-025-06750-x)

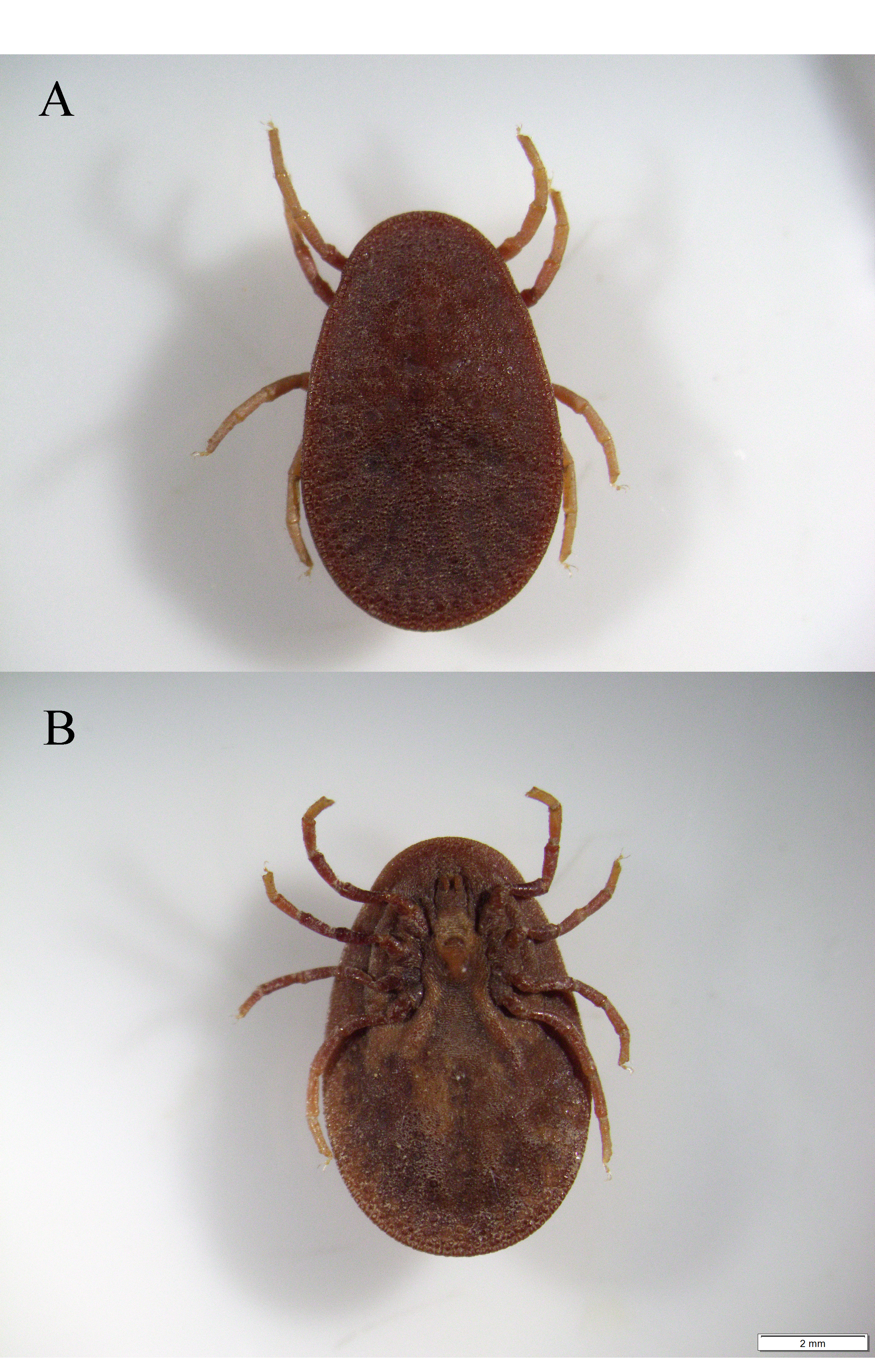

Supplement: Supplementary file 1 — Additional file 1: Fig. S1. Morphological characteristics of the Argas persicus tick from Xinjiang. The capitulum is not visible viewing from above. Body oval and flat dorso-ventrally. Discs are oval or rounded with different sizes. A lateral sutural line is present, with distinct rectangular squares around the entire body margin [file 13071_2025_6750_MOESM1_ESM.jpg]
